# Long Non-coding RNA LINC01094 Promotes the Development of Clear Cell Renal Cell Carcinoma by Upregulating SLC2A3 via MicroRNA-184

**DOI:** 10.3389/fgene.2020.562967

**Published:** 2020-09-23

**Authors:** Haifei Xu, Xiaolin Wang, Jiacheng Wu, Hao Ji, Zhigang Chen, Haifeng Guo, Jianquan Hou

**Affiliations:** ^1^Department of Urology, The First Affiliated Hospital of Soochow University, Suzhou, China; ^2^Department of Urology, Nantong Tumor Hospital, Nantong, China

**Keywords:** clear cell renal cell carcinoma, long non-coding RNA, LINC01094, microRNA-184, solute carrier family 2 facilitated glucose transporter member 1

## Abstract

Clear cell renal cell carcinoma (ccRCC) is the most common subtype of RCC. Compelling evidence has highlighted the crucial role of long non-coding RNA (lncRNA) in ccRCC. Our current study aims to explore the regulatory mechanism of LINC01094 in the development of ccRCC. Dual-luciferase reporter experiment verified the targeting relationship among miR-184, LINC01094, and SLC2A3. Furthermore, the interaction between LINC01094 and miR-184 was confirmed by RNA immunoprecipitation (RIP) and RNA pull-down. Biological behaviors of ccRCC cells were investigated through cell counting kit-8 (CCK8), scratch test, Transwell, and flow cytometry. The effect of SLC2A3 on the tumorigenicity of nude mice was evaluated *in vivo*. In ccRCC cells and clinical tissues, LINC01094 and SLC2A3 were highly expressed while miR-184 was lowly expressed. Besides, miR-184 was verified to be a direct target of LINC01094. Silencing LINC01094, up-regulating miR-184, or reducing SLC2A3 inhibited the growth, migration, and invasion of ccRCC cells. Tumor growth was suppressed by silenced LINC01215 via reducing the expression of SLC2A3 via miR-184. Taken together, silencing LINC01094 inhibited SLC2A3 expression by up-regulating miR-184, thereby inhibiting the development of ccRCC.

## Introduction

Clear cell renal cell carcinoma (ccRCC) has a high rate of mortality affecting more than 40% of patients, rating as the third leading urological cancer only after prostate cancer and bladder cancer (Szabo et al., 2016). Early diagnosis and nephrectomy are considered as the most effective measure to cure ccRCC at its early stage (Lou et al., 2017). However, the diagnosis of ccRCC is yet confronted with problems such as lacking biomarkers. Therefore, more effective therapy for improving the diagnosis and prognosis of patients with ccRCC is a prerequisite to developing promising molecular biomarkers (Qin et al., 2019).

Notably, long non-coding RNAs (lncRNAs) have been shown to play crucial roles in cancer biology (Yu et al., 2012). Evidence from previous works shows that lncRNAs are always aberrantly expressed in human urologic cancers (Martens-Uzunova et al., 2014). Besides, lncRNAs signature shows prognostic value in ccRCC (Qu et al., 2018). LncRNA NONHSAT123350 participates in the pathogenesis of ccRCC (Liu et al., 2016). LncRNA MRCCAT1 is a pro-tumor gene that can promote metastasis of ccRCC by activating the p38-MAPK signaling (Li et al., 2017). It has been reported that lncRNA LINC01094 exhibits high expression in tissues of ccRCC while deleting lncRNA LINC01094 exerts inhibition on the growth and metastasis of ccRCC cells (Jiang et al., 2020). However, the detailed mechanism underlying LINC01094 remains to be further elucidated. According to the microarray-based analysis in our study, LINC01094 in tumor samples was differentially expressed. Emerging evidence implicates that lncRNAs can bind to microRNAs (miRNAs; Du et al., 2016). Whilst miRNAs play pivotal roles during tumor initiation and progression (Zhang et al., 2018). It has also been suggested that aberrantly expressed miRNAs are related to the occurrence and development of ccRCC through participating in critical biological processes (Zhao et al., 2018). For example, miR-184 can exert certain effects on the metastasis and proliferation of ccRCC cells (Huang et al., 2016). Moreover, miR-184 possesses an inhibiting role in the development of ccRCC (Zhou et al., 2010). Hence, several web-available databases exploring miRNA-mRNA interaction illustrated SLC2A as a potential target of miR-184. Besides, high expression of solute carrier family 2 facilitated glucose transporter member 1 (SLC2A) is related with poor prognosis in many cancers (Kim et al., 2019). Thus, based on the above-described findings, we aim to determine the role of LINC01094/miR-148/SLC2A regulatory network in ccRCC, in a bid to find a promising strategy for ccRCC treatment.

## Materials and Methods

### Bioinformatics Analysis

The ccRCC-related microarray data were obtained from the Gene Expression Omnibus (GEO) database^1^, and differential analysis was performed on the retrieved datasets of GSE53757 (miRNA) and GSE53757 (gene). The affy package of R language was used for background correction and normalization pre-processing while the limma package for differentially expressed gene screening. The corrected *p*-value was expressed as adj.*P*.Val < 0.05 and the screening threshold was set as | log2FoldChange (FC)| > 1.0 and adj.*P*. Val < 0.05, followed by the drawing of the heat map of differentially expressed genes. The LncATLAS^2^ database was used for subcellular localization of lncRNA, then the RNA22 database^3^ was employed to predict the possible binding sites between miRNAs and differentially expressed lncRNAs. Next, the target genes of miRNA were predicted using mirDIP^4^, RNA22, miRWalk^5^, and starBase^6^. Additionally, Venn online analysis tool was used to analyze the intersections and plot the Venn diagrams^7^.

### Study Subjects

The ccRCC tissues and adjacent normal kidney tissues (> 5 cm away from the tumor site) were collected from patients who received radical nephrectomy in the Urology Department of Nantong Tumor Hospital from June 2015 to June 2019. One part of the resected tissue was restored at a −80°C, and the other part was fixed in 10% neutral formalin, embedded in paraffin, and cut into 4 μm sections. Among the patients, 37 were males and 19 were females, aging from 31 to 80 years old. According to the American Joint Committee on Cancer (AJCC) staging, there were 10 cases in stage I, 25 in stage II, and 21 in stage III. Fuhrman grading represents 27 patients in G1 + G2 while 29 in G3 + G4. Vascular invasion was recorded in 19 patients, whereas 37 cases did not exhibit vascular invasion. Lymph node metastasis was observed in the 25 patients but 31 cases did not exhibit lymph node metastasis. Inclusion criteria for the ccRCC patients were: (1) diagnosed as ccRCC; (2) had no combined malignant tumors; (3) had no tumors in the kidney, such as renal cysts and renal hamartomas. The diameter of the tumor was based on the largest diameter of the tumor reported by pathological reports and imaging.

### Immunohistochemistry

The sections were dehydrated and stained through the conventional immunohistochemical staining method. Then the primary antibody rabbit anti-SLC2A3 (ab15311, 1: 50) and secondary antibody immunoglobulin G (IgG; ab150083, 1: 100), which were purchased from Abcam, Inc. (Cambridge, United Kingdom), were incubated with the sections, respectively. The normal saline was used as the negative control (NC) of the primary antibody. After developed by diaminobenzidine (DAB) and stained with hematoxylin for 5 min, five visual fields were randomly selected and photographed under a microscope. And the number of positively stained cells was counted and presented as %. Besides, the percentage of SLC2A3 positive cells < 15% was determined as a negative group, while > 15% was a positive group.

### Cell Culture

Three ccRCC cell lines (769-P, Caki-1, and 786-O) and human normal proximal tubule epithelial cells HK-2 were obtained from the Cell Bank of the Chinese Academy of Sciences^8^. HK-2 cell line was cultured in high glucose Dulbecco’s modified eagle medium (DMEM; 12800017, GIBCO, Carlsbad, CA, United States) containing 10% Fetal bovine serum (FBS, 26140079, GIBCO, United States) and 1% penicillin-streptomycin. Then the 769-P, Caki-1, and 786-O cells were cultured in RPMI1640 medium supplemented with 10% FBS and 1% penicillin-streptomycin. All the above-mentioned cells were incubated in an incubator (BB15, Thermo Fisher Scientific, Inc., Waltham, MA, United States) at 37°C with 5% CO_2_. The culture medium was renewed every 24 h and the cells were subcultured every 72 h. The expression of LINC01094 in each cell line was measured by reverse transcription-quantitative polymerase chain reaction (RT-qPCR), and the cell line with the highest expression was selected for subsequent experiments.

### Cell Grouping and Transfection

The cells were seeded in six-well plates 24 h before transfection. After achieving 79% of the cell confluence, cell transfection was performed under the instructions of the Lipofectamine2000 kit (11668019, Thermo Fisher Scientific, Inc., Waltham, MA, United States). The cells were transfected with short hairpin RNA against LINC01094 (sh-LINC01094), LINC01094 overexpression vector (oe-LINC01094), miR-184 mimic plasmid, miR-184 inhibitor plasmid, shRNA targeting SLC2A3 (sh-SLC2A3), SLC2A3 overexpression vector (oe-SLC2A3), and the corresponding NC (sh-L-NC, oe-L-NC, NC mimic, NC inhibitor, sh-S-NC, and oe-S-NC). Meanwhile, the interfering efficiency of shRNA (sh-LINC01094#1, sh-LINC01094#2, sh-LINC01094#3, sh-SLC2A3#1, sh-SLC2A3#2, and sh-SLC2A3#3) was detected by the RT-qPCR. The shRNA with the lowest expression of LINC01094 or SLC2A3 was selected for further analysis. All plasmids, vectors, sequences, viral package, and titer detection were entrusted to the GeneChem Biotech (Shanghai, China).

### RT-qPCR

The total RNA was extracted from collected tissues or cells using the TRIzol (Invitrogen, Carlsbad, CA, United States) which was pre-cooled at 4°C. Following after, RNA was reversely transcribed into complementary DNA (cDNA) using the PrimeScript reverse transcription kit (TaKaRa, Tokyo, Japan) and cDNA reverse transcription kit (K1622, Reanta Biotechnology, Beijing, China). Afterward, the fluorescent quantitative PCR was conducted on the ABI7500 fluorescent quantitative PCR instrument (Applied Biosystems, Foster City, CA United States) under the instructions of SYBR^®^ Premix Ex TaqTM II Kit (TaKaRa, Tokyo, Japan). The reaction system consisted of 10 μL SYBR^®^ Premix, 0.6 μL upstream primer, 0.6 μL downstream primer, 1 μL ROX Reference Dye (50 ×), 2 μL total cDNA template, and 6.8 μL ddH2O. The amplification conditions were as follows: pre-denaturation at 95°C for 30 s, followed by denaturation at 95°C for 50 s, annealing at 60°C for 30 s, and extension at 72°C for 30 s, for a total of 35 cycles. With U6 and β-actin as internal references, the relative expression of the target genes was calculated using the relative quantification method (2^–△ △ CT^ method), and the formula was as follows: ΔΔCt = ΔCt_experimental group_ − ΔCt_control group_, ΔCt = Ct_target gene_ − Ct_internal reference_. The experiment was repeated in triplicates. Primers used in this experiment are listed in Table 1.

**TABLE 1:** Primer sequences for RT-PCR.

Gene (Human)		Primer Sequence
LINC01094	Forward	5′-TCCCTTCCACAGAGAAGGCT-3′
	Reverse	5′-AGGTTGACACATCTCGCCTG-3′
miR-184	Forward	5′-GCTTGGCCCTGACCTTATGT-3′
	Reverse	5′-TTCCTGTCGTCTTGCTTCCC-3′
SLC2A3	Forward	5′-GCTCTTTCCAATTTGGCTA-3′
	Reverse	5′-GCTTCCTCATTACCTTCTT-3′
β-actin	Forward	5′-TCCTGTGGCATCCACGAAACT-3′
	Reverse	5′-GAAGCATTTGCGGTGGACGAT-3′
U6	Forward	5′-CTCGCTTCGGCAGCACA-3′
	Reverse	5′-ACGCTTCACGAATTTGCGT-3′

*RT-qPCR, reverse-transcription quantitative polymerase chain reaction; LINC01094, Long non-coding RNA LINC01094; miR-184, microRNA-184; SLC2A3, solute carrier family 2 member 3.*

### Western Blot Analysis

The total protein was extracted from cells or tissues with radio-immunoprecipitation assay (RIPA) lysate containing phenylmethylsulfonyl fluoride (PMSF; R0010, Beijing Solarbio Science & Technology, Co., Ltd., Beijing, China). Then, the protein concentration was measured using a bicinchoninic acid (BCA) kit (20201ES76, Yeasen Company, Shanghai, China). After the protein was separated by polyacrylamide gel electrophoresis, the protein was transferred onto a polyvinylidene fluoride membrane. The membrane was blocked with 5% bovine serum albumin (BSA) for 1 h at room temperature followed by incubation with diluted rabbit primary SLC2A3 antibody (ab15311, 1: 1000, Abcam, Inc., Cambridge, United Kingdom) overnight at 4°C. The next day, the membrane was incubated with horseradish peroxidase (HRP)-labeled secondary rabbit IgG antibody (1: 5000, ProteinTech, Inc., Rosemont, IL, United States) at room temperature for 1 h. Subsequently, after washing with TBST three times (10 min per time), the protein bands were developed and visualized. Finally, the relative protein content was expressed by the ratio of the gray value of the target band to the gray value of glyceraldehyde-3-phosphate dehydrogenase (GAPDH; 1: 5000, 10494-1-AP, ProteinTech, Inc., United States), which were analyzed using Quantity One v4.6.2 software analysis.

### Dual-Luciferase Reporter Assay

The 3′-untranslated regions (3′-UTR) of LINC01094 or SLC2A3 mRNA containing wild type (WT) or mutant (MUT) binding site was artificially synthesized and inserted into the pmiR-RB-REPORT^TM^ plasmid (RiboBio, Co., Ltd., Guangzhou, China) using restriction endonuclease. Meanwhile, the empty plasmid was taken as a negative control. The correctly sequenced luciferase reporter plasmids WT and MUT were co-transformed with NC mimic or miR-184 mimic into HEK293T cells, respectively. After 48 h of transfection, the cells were collected and lysed and centrifuged for 3–5 min followed by the collection of supernatant. Then, the relative luciferase unit (RLU) was evaluated on a dual-luciferase reporting analysis system (Promega Corporation, Madison, WI, United States) using a Renilla luciferase detection kit (YDJ2714, Shanghai Yuduo Biotechnology, Co., Ltd., Shanghai, China). The relative fluorescence activity was expressed as the ratio of the Renilla RLU value to the firefly RLU value.

### RNA Pull-Down

Cells were transfected with 50 nM biotinylated WT-bio-miR-184 and MUT-bio-miR-184 (Wuhan Genecreate Biological Engineering, Co., Ltd., Wuhan, China). After 48 h of transfection, cells were collected and washed with phosphate buffer saline (PBS). Then the cells were incubated in specific lysis buffer (Ambion, Austin, TX, United States) for 10 min. Then the lysates were incubated with M-280 streptavidin magnetic beads (S3762, Sigma-Aldrich, St Louis, MO, United States) which were pre-coated with RNase-free BSA and yeast tRNA (TRNABAK-RO, Sigma-Aldrich, St Louis, MO, United States) at 4°C overnight. Subsequently, the magnetic beads were washed twice with pre-cooled lysis buffer, three times with low salt buffer, and once with high salt buffer. At last, the bound RNA was purified by the TRIzol, and RT-qPCR was used to detect the enrichment of LINC01094. The experiment was repeated three times.

### RNA Immunoprecipitation (RIP) Assay

The RIP kit (Merck Millipore, Billerica, MA, United States) was used to detect the combination of LINC01094 and Ago2. Cells were lysed with an equal volume of RIPA lysis buffer (P0013B, Beyotime Biotechnology, Co., Shanghai, China) on ice for 5 min and then centrifuged at 14,000 rpm at 4°C for 10 min followed by the collection of supernatant. Part of the supernatant was taken as input, whereas, another part was incubated with antibodies for co-precipitation. The samples were placed on a magnetic base to collect the magnetic bead-protein complex. After that, the samples and input were detached with proteinase-K to extract RNA for subsequent PCR detection. The antibody used in the RIP assay was rabbit anti-Ago2 (ab186733, 1: 50, Abcam) which was mixed at room temperature for 30 min, and rabbit anti-IgG (ab109489, 1: 100, Abcam) was used as NC. The experiment was repeated three times independently.

### Fluorescence *in situ* Hybridization (FISH) Assay

The cells were seeded on a coverslip in a 24-well plate at a density of 6 × 10^4^ cells/well. Once the cell confluence reached about 60–70%, the cells were washed with PBS and then fixed with 4% paraformaldehyde containing 0.5% Triton X-100 for 10 min at room temperature. Afterward, a total of 20 μL of pre-hybridization solution (BREA-106, Beijing Biocreative Technology, Co., Ltd., Beijing, China) was added to each well to block cells at 37°C for 30 min followed by the incubation with the Stellaris RNA FISH (Biosearch Technologies, Petaluma, CA, United States) probe hybridization solution containing LINC01094 probe at 37°C overnight in the dark. Subsequently, cells were stained with 4,6-diamino-2-phenyl indole (DAPI) staining solution for 10 min devoid of light. The slides were then washed three times with PBS (5 min per time), and then sealed using an anti-fluorescence quenched mounting medium (BIH0252, BioRike, Tokyo, Japan). Finally, a fluorescence microscope (Olympus, Tokyo, Japan) was applied to observe and photograph the cells in five randomly selected view fields. The experiment was repeated three times independently.

### Cell Counting Kit-8 (CCK8) Assay

The cells in the logarithmic growth phase were seeded in 96-well plates with a density of 5 × 10^3^ cells per well and cultured with 5% CO_2_ at 37°C. After 24, 48, and 72 h of culture, a total of 10 μL of CCK-8 solution (CA1210-100, Beijing solarbio science & technology, Co., Ltd., Beijing, China) was added to each well for incubation in the incubator for another 2 h. Subsequently, the optical density (OD) of each well at 450 nm was measured using a microplate reader (BIO-RAD 680, Bio-Rad Laboratories, Inc., Hercules, CA, United States). The cell proliferation curve was plotted lastly.

### Scratch Test

The transfected cells of each group were incubated at 37°C in 5% CO_2_ for 24 h. Then a 10 μL pipette tip was used to make scratches on the monolayer cells. After removing the exfoliated cells which were induced by pipette tip, a serum-free medium was added. The cells were then observed and photographed at 0 and 24 h under an inverted microscope. The experiment was repeated three times.

### Transwell Assay

The pre-cooled serum-free DMEM medium-diluted Matrigel (40111ES08, Yeasen Company, Shanghai, China; Matrigel: DMEM = 1:2) was added to cover the apical chamber of Transwell chamber (3413, Beijing Unique biotechnology, Co., Ltd., Beijing, China), which was then placed in an incubator at 37°C for 4–5 h to solidify the Matrigel. Then, the transfected cells were diluted with 100 μL of serum-free medium to prepare a cell suspension with a concentration of about 1 × 10^6^ cells/mL followed by inoculation into the apical chamber. A total of 500 μL of DMEM medium containing 20% FBS was added to the basolateral chamber. After incubation at 37°C for 24 h, the Transwell chambers were washed twice with PBS and the non-invaded cells were wiped off with a cotton ball. As for the invaded cells, they were fixed with 5% glutaraldehyde at 4°C, followed by staining by 0.1% crystal violet for 5 min. With five view fields randomly selected, the cells were observed and photographed under an inverted fluorescence microscope (TE2000, Nikon, Beijing, China). The number of invaded cells in each group was counted with the mean value. The experiment was repeated three times.

### Flow Cytometry

After cell transfection for 48 h, the cells were detached with 0.25% trypsin (without ethylene diamine tetraacetic acid), centrifuged, and collected in a flow tube. According to the instructions of Annexin-V-FITC Apoptosis Detection Kit (556547, Becton, Dickinson and Company), to prepare Annexin-V/PI staining solution, the Annexin-V-FITC, PI and HEPES buffer solution were mixed at a ratio of 1:2:50. Afterward, a total of 1 × 10^6^ cells were resuspended in 100 μL of the staining solution, followed by incubation for 15 min at room temperature. Then 1 mL of HEPES buffer was added. The cell apoptosis was detected using flow cytometry (Bio-Rad ZE5, Bio-Rad Laboratories, Inc., Hercules, CA, United States). The maximum absorption wavelength of FITC was 488 nm, the emission wavelength was 525 nm, while the maximum absorption and emission wavelengths of the PI-DNA complex were 535 nm and 615 nm, respectively. The experiment was repeated three times independently.

### Xenograft Tumor in Nude Mice

Twenty-four specific pathogen-free (SPF) male nude mice (aging 4 weeks and weighing 20–22 g; Shanghai Slac Laboratory Animal, Co., Ltd., Shanghai, China) were randomly divided into four groups with six mice each group. A total of 2 × 10^6^ cells, which were stably transfected with oe-LINC01094, sh-LINC01094, or the relevant NC, were responded in the mixture of normal saline (50 μL) and Matrigel Matrix (50 μL). Thereafter, the cell suspensions were injected into the underarm of nude mice. After 7, 14, 21, and 28 days, the mice were euthanized; the xenograft tumors were collected, weighed, and compared, respectively.

### Statistical Analysis

Statistical Package for the Social Sciences (SPSS) 21.0 (IBM SPSS Statistics, Armonk, NY, United States) was used for statistical analysis. The measurement data were expressed by the mean ± standard deviation (SD). A paired *t*-test was used to compare the cancer tissues and adjacent normal tissues while the unpaired data were analyzed by unpaired *t*-test. One-way analysis of variance (ANOVA) was used to compare data between multiple groups, and Tukey’s was used for *post hoc* tests. The cell viability of each group at different time points was compared using two-way ANOVA. Tumor volume data at multiple time points were compared using repeated-measurement ANOVA followed by Bonferroni’s *post hoc* test. Then, Pearson’s correlation analysis was applied to observe the correlation of indicators. Enumeration data were expressed by number of cases and percentage, and analyzed by chi-square test. The *p* < 0.05 was considered to be statistically significant.

## Results

### LINC01094 Was Highly Expressed in ccRCC Tissues and Cells

**FIGURE 1:**
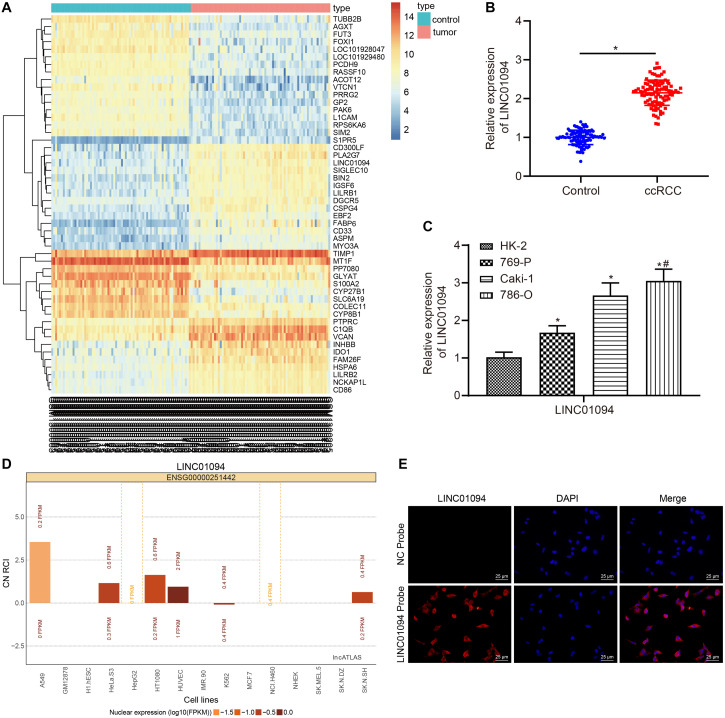
High expression of LINC01094 was found in clear cell renal cell carcinoma (ccRCC) tissues and cells. **(A)** Heat map of 50 differential expressed genes in the ccRCC-related microarray dataset GSE53757. The *X*-axis represents the sample number and the *Y*-axis represents the differentially expressed gene. Each rectangle in the Figure corresponded to the expression of a gene in a sample. **(B)** The expression of LINC01094 in clinical ccRCC and adjacent normal tissues detected by reverse transcription-quantitative polymerase chain reaction (RT-qPCR). **(C)** The expression of LINC01094 in different cell lines evaluated by RT-qPCR. **(D)** The subcellular location of LINC01094 according to LncATLAS database. CN RCI refers to the ratio of long non-coding RNA (lncRNA) expression in the cytoplasm and in the nucleus of a cell line. FPKM, Fragments per kilobase of exon model per million mapped fragments. **(E)** The subcellular localization of LINC01094 measured by FISH (400 ×). LINC01094 showed red fluorescence and the nucleus showed blue fluorescence. * *p* < 0.05 *vs*. the control group or HK-2, # indicated the cell line with the highest expression of LINC01094. The data are measurement data and expressed as mean ± SD. Paired *t*-test is performed for comparison between ccRCC tissues and adjacent normal tissues. One-way ANOVA is used to compare data among multiple groups, and Tukey’s was used for *post hoc* tests.

The differentially expressed genes associated with ccRCC were analyzed using the R language and the results showed that there were 886 differentially expressed genes in the microarray dataset GSE53757. Besides, the heat map showed the 50 genes with the maximum changes in the microarray dataset GSE53757 (Figure 1A). Afterward, we noticed that the LINC01094 expression in ccRCC tissues was higher than that in the adjacent normal tissues. Then, the results of RT-qPCR confirmed the higher expression of LINC01094 in clinical ccRCC tissues compared with the adjacent normal tissue (*p* < 0.05; Figure 1B). Subsequently, the analysis of the correlation between LINC01094 and clinicopathological characteristics was conducted in of 56 ccRCC cases, which revealed that the number of cases with high expression of LINC01094 was far more than that with low expression of LINC01094 (all *p* < 0.05). Additionally, the expression of LINC01094 was not related with the patient’s gender, age, and tumor size (all *p* > 0.05); however, it was related to the TNM stage, Fuhrman grade, vascular invasion, and lymph node metastasis (all *p* < 0.05; Table 2). Therefore, we speculated that LINC01094 was one of the key molecules in the development of ccRCC. After that, the expression of LINC01094 was evaluated in three ccRCC cell lines (769-P, Caki-1, and 786-O) and human normal proximal tubular epithelial cells HK-2 using RT-qPCR, while 786-O cells, which exhibited the highest expression, were selected for subsequent experiments (both *p* < 0.05; Figure 1C). The subcellular localization of lncRNAs is the primary determinant of their molecular functions (Carlevaro-Fita and Johnson, 2019). The subcellular fate of lncRNAs has the potential to provide new insights into their biogenesis and specialized functions (Chen, 2016). Then, according to the LncATLAS database, the expression of LINC01094 was higher in the cytoplasm (Figure 1D), while FISH confirmed the up-regulated expression of LINC01084 in the cytoplasm of cancer cells (Figure 1E). Taken together, LINC01094, which was located in the cytoplasm, was highly expressed in ccRCC tissues and cells.

**TABLE 2:** Relationship between the expression of LINC01094 and the clinicopathological characteristics of clear cell renal cell carcinoma (ccRCC).

Clinicopathological characteristics	Cases	Expression of LINC01094		*P* value
		Low expression	High expression	
Gender				0.7808
Male	36	19(52.78%)	17(47.22%)	
Female	20	9(45.00%)	11(55.00%)	
Age				0.7875
≤ 60	31	15(51.61%)	17(48.39%)	
> 60	25	13(48.00%)	11(52.00%)	
Size				0.7848
≤ 4 cm	22	10(45.45%)	12(54.55%)	
> 4 cm	34	18(52.94%)	16(47.06%)	
TNM stage				0.0052
I+II	35	23(65.71%)	12(34.29%)	
III+IV	21	5(23.81%)	16(76.19%)	
Fuhrman grading				0.0315
G1+G2	27	18(66.67%)	9(33.33%)	
G3+G4	29	10(34.48%)	19(65.52%)	
Vascular invasion				0.0041
Yes	19	4(21.05%)	15(78.95%)	
No	37	24(64.86%)	13(35.14%)	
Lymph node metastasis				0.0403
Yes	11	2(18.18%)	9(81.82%)	
No	45	26(57.78%)	19(42.22%)	

*The data were measurement data, and compared using chi-square test, sample size N = 56, p < 0.05 was considered to be statistically significant.*

### Silencing LINC01094 Inhibited ccRCC Progression

**FIGURE 2:**
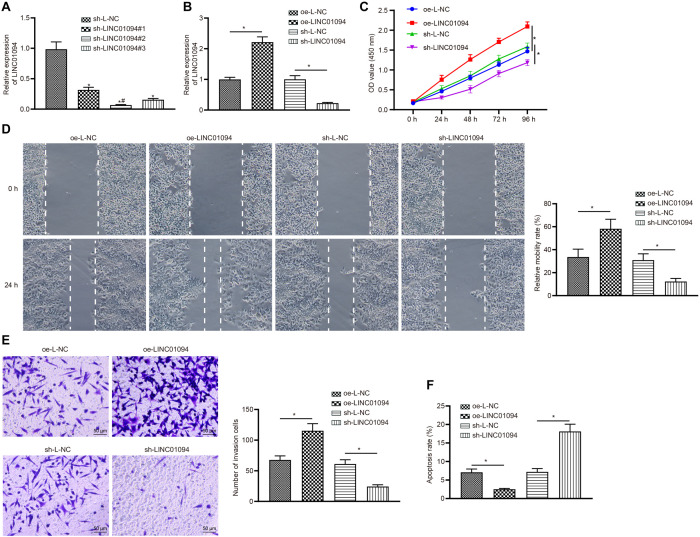
Silencing LINC01094 can inhibit the progression of clear cell renal cell carcinoma (ccRCC). **(A)** The interference efficiency of LINC01094 detected by reverse transcription-quantitative polymerase chain reaction (RT-qPCR). **(B)** The expression of LINC01094 in cells after transfection evaluated by RT-qPCR. **(C)** The cell viability of 786-O cells measured by cell counting kit-8 (CCK8). **(D)** The detection of the migration ability of 786-O cells in each group via scratch assay (100 ×). **(E)** The invasion ability of 786-O cells detected by Transwell assay (200 ×). **(F)** Cell apoptosis detection by flow cytometry. * *p* < 0.05 *vs.* the oe-L-NC group or the sh-L-NC group. # indicated the cell line with the lowest expression of LINC01094. The data are measurement data and expressed as mean ± SD. Paired *t*-test is performed for comparison between ccRCC tissues and adjacent normal tissues. One-way ANOVA is used to compare data among multiple groups, followed by Tukey’s *post hoc* tests. Data at different time points are compared using two-way ANOVA.

To investigate the effects of LINC01094 on the occurrence and development of ccRCC, three silent interference sequences for LINC01094 were designed. Then the silent interference efficiency was detected by the RT-qPCR, which showed that the sh-LINC01094#2 sequence exhibited the highest silent interference efficiency (all *p* < 0.05; Figure 2A). Therefore, the sh-LINC01094#2 sequence was selected for subsequent experiments. Accordingly, our results of RT-qPCR revealed that the transfection with oe-LINC01094 increased the expression of LINC01094, while sh-LINC01094 led to the opposite tendency (all *p* < 0.05; Figure 2B). Then, CCK8 was conducted to detect the viability of ccRCC cells in each group. It was found that overexpression of LINC01094 promoted the growth of 786-O cells, but silencing LINC01094 inhibited it (both *p* < 0.05; Figure 2C). Moreover, the migration and invasion abilities were accessed by Scratch assay and Transwell assay, respectively, which showed that the migration and invasion of 786-O cells were promoted by oe-LINC01094 but suppressed by sh-LINC01094 (all *p* < 0.05; Figures 2D,E). According to the flow cytometry, the transfection of oe-LINC01094 could decrease the apoptosis rate of 786-O cells while sh-LINC01094 significantly increased the cell apoptosis rate (all *p* < 0.05; Figure 2F). Altogether, the above results suggested that overexpression of LINC01094 promoted the proliferation, migration, and invasion, but inhibited cell apoptosis of 786-O cells.

### LINC01094 Bound to miR-184 in ccRCC

**FIGURE 3:**
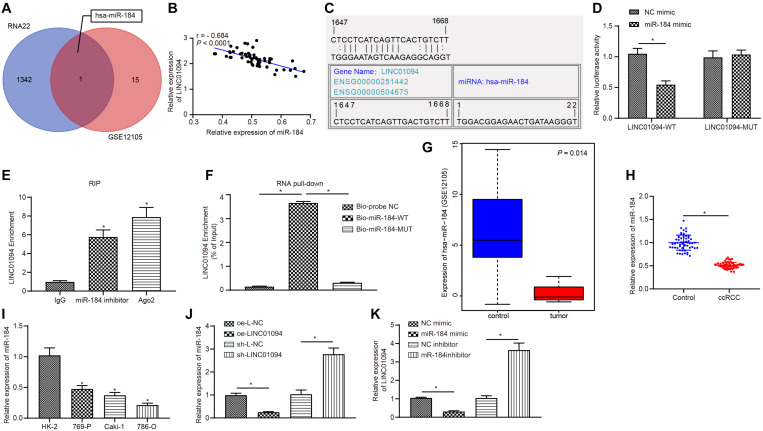
LINC01094 could regulate miR-184 expression through binding to miR-184. **(A)** Bioinformatics prediction on microRNAs (miRNAs) that might be regulated by LINC01094. **(B)** Analysis of the correlation between miR-184 and LINC01094. **(C)** RNA22 prediction of the targeting relationship. **(D)** The targeting relationship between LINC01094 and miR-184 verified by dual luciferase reporter assay. **(E)** The enrichment of LINC01094 measured by RNA immunoprecipitation (RIP). **(F)** The binding of miR-184 to LINC01094 evaluated by RNA pull-down assay. **(G)** Analysis on the GSE12105 microarray dataset. **(H)** miR-184 expression in clinical clear cell renal cell carcinoma (ccRCC) and adjacent normal tissues measured by reverse transcription-quantitative polymerase chain reaction (RT-qPCR). **(I)** miR-184 expression in different cell lines measured by RT-qPCR. **(J)** RT-qPCR detection of the relative expression of miR-184 in each group of cells. **(K)** RT-qPCR detection of the relative expression of LINC01094 in each group of cells. * *p* < 0.05 *vs.* the NC mimic group or immunoglobulin G (IgG) group or Bio-probe NC group or control group or HK-2 or oe-L-NC group or sh-L-NC group or NC inhibitor group. The data are measurement data and expressed as mean ± SD. Paired *t*-test is performed for comparison between ccRCC tissues and adjacent normal tissues. One-way ANOVA is used to compare data among multiple groups, followed by Tukey’s *post hoc* tests. Pearson correlation analysis is used to observe the correlation of indicators.

The RNA22 database was used to predict the possible miRNAs regulated by LINC01094, with 1343 miRNAs obtained. Besides, 16 differentially expressed miRNAs were screened out in the miRNA microarray dataset. When we intersected the miRNA results predicted by RNA22 with the 16 differentially expressed miRNAs, there was only one miRNA, hsa-miR-184 (Figure 3A). Pearson correlation analysis (Figure 3B) showed that the level of miR-184 in ccRCC tissues was negatively correlated with LINC01094 expression (both *p* < 0.0001). Therefore, it was speculated that miR-184 expression was regulated by LINC01094. Furthermore, according to the RNA22 database, there exist binding sites between the miR-184 and 3′UTR of LINC01094 (Figure 3C). Meanwhile, a dual-luciferase reporter assay was performed to verify the targeting relationship between LINC01094 and miR-184. Compared with the cells transfected with NC mimic, the luciferase activity of the cells co-transfected with both miR-184 mimic and WT-LINC01094 was lower than that of the cells treated with NC, whereas, cells co-transfected with miR-184 mimic and MUT-LINC01094 did not change significantly (Figure 3D). Subsequently, the RIP assay was adopted to detect the enrichment of LINC01094 in each group, which showed that the enrichment levels of LINC01094 in cells treated with miR-184 inhibitor or Ago2 were higher than that of IgG-treated cells, and the enrichment level of LINC01094 was the highest in Ago2-treated cells (all *p* < 0.05; Figure 3E). Also, the results of the RNA pull-down assay further verified that binding between LINC01094 and miR-184 (both *p* < 0.05; Figure 3F).

According to the microarray dataset GSE12105, the expression of miR-184 was down-regulated in ccRCC (Figure 3G). Then, RT-qPCR was performed to measure the miR-184 expression on the collected ccRCC tissues and the adjacent normal tissues, which showed that the expression of miR-184 in ccRCC tissues was much lower than that in adjacent normal tissues (*p* < 0.05; Figure 3H). Furthermore, compared with HK-2 cells, miR-184 expression was much lower in Caki-1, 786-O, and 769-P cells, and 786-O cells showed the lowest miR-184 expression. Therefore, the 786-O cell line was selected for subsequent experiments (both *p* < 0.05; Figure 3I). The above results indicated that miR-184 was poorly expressed in ccRCC tissues and cells.

Additionally, RT-qPCR was also conducted to detect the expression of LINC01094 and miR-184 in cells of each group after transfection. The results showed that the miR-184 expression was decreased in cells after oe-LINC01094 treatment, while increased after sh-LINC01094 treatment (both *p* < 0.05; Figure 3J). Whilst compared with the NC mimic, the transfection of miR-184 mimic decreased the levels of LINC01094, but the levels of LINC01094 were increased by the treatment of miR-184 inhibitor compared with the NC inhibitor (all *p* < 0.05; Figure 3K). In a word, the results indicated that LINC01094 could bind to miR-184, and overexpressed LINC01094 inhibited miR-184 expression.

### LINC01094 Promoted the Progression of ccRCC by Down-Regulating miR-184

**FIGURE 4:**
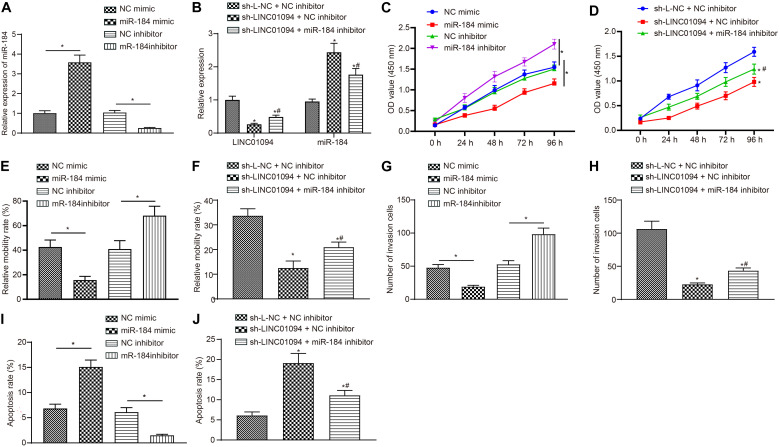
LINC01094 affects clear cell renal cell carcinoma (ccRCC) progression by regulating miR-184 expression. **(A,B)** The expression of LINC01094 and miR-184 measured using reverse transcription-quantitative polymerase chain reaction (RT-qPCR). **(C,D)** The cell viability of ccRCC cells measured using cell counting kit-8 (CCK8). **(E,F)** The migration ability of ccRCC cells examined by scratch test. **(G,H)** The invasion ability of ccRCC cells in each group detected by Transwell assay (200 ×). **(I,J)** The apoptosis of ccRCC cells in each group accessed by flow cytometry. * *p* < 0.05 *vs.* the NC mimic group or NC inhibitor group or sh-L-NC + NC inhibitor group, # *p* < 0.05 *vs.* sh-LINC01094 + NC inhibitor group. The data are measurement data and expressed as mean ± SD. Paired *t*-test is performed for comparison between ccRCC tissues and adjacent normal tissues. One-way ANOVA is used to compare data among multiple groups, followed by Tukey’s *post hoc* tests. Data at different time points are compared using two-way ANOVA.

At first, ccRCC cells were transfected with NC mimic, miR-184 mimic, NC inhibitor, and miR-184 inhibitor plasmids. Then RT-qPCR was carried out to test the levels of miR-184, which showed that miR-184 mimic successfully up-regulated the levels of miR-184. Meanwhile, miR-184 expression was inhibited in cells treated with miR-184 inhibitor (both *p* < 0.05; Figure 4A). The results of CCK8 (Figure 4C), scratch test (Figure 4E), Transwell assay (Figure 4G), and flow cytometry (Figure 4I) showed that the transfection of miR-184 mimic markedly lowered the proliferation, migration, and invasion abilities of ccRCC cells, but significantly promoted the cells apoptosis, while the treatment of miR-184 inhibitor led to the opposite trends (all *p* < 0.05).

To study the effect of LINC01094 on the function of miR-184, the cells were transfected with sh-L-NC + NC inhibitor, sh-LINC01094 + NC inhibitor, and sh-LINC01094 + miR-184 inhibitor, respectively. Then, the expressions of LINC01094 and miR-184 were evaluated by RT-qPCR (all *p* < 0.05; Figure 4B). Compared with cells transfected with sh-L-NC + NC inhibitor, other treatments significantly decreased the expression of LINC01094 but increased that of miR-184. In comparison to the treatment of sh-LINC01094 + NC inhibitor, after the transfection with sh-LINC01094 + miR-184 inhibitor, the level of LINC01094 increased while the level of miR-184 was decreased. CCK8 assay (Figure 4D), scratch test (Figure 4F), Transwell assay (Figure 4H), and flow cytometry (Figure 4J) were subsequently conducted. When transfected with sh-LINC01094 + NC inhibitor, the viability, migration, and invasion abilities of cells were reduced, but the apoptosis rate of the cells was increased. Furthermore, the treatments of sh-LINC01094 + miR-184 inhibitor led to up-regulated viability, migration, and invasion abilities of cells, but down-regulated cell apoptosis rate (all *p* < 0.05). The above-mentioned results indicated that the effects of sh-LINC01094 on the viability, migration, invasion, and apoptosis of ccRCC cells could be reversed by miR-184 inhibitor. As revealed in Supplementary Figure S1, same results were also observed in 767-P cell line that LINC01094 promoted ccRCC progression through down-regulation of miR-184.

### SLC2A3, a Target of miR-184, Promoted the Development of ccRCC

**FIGURE 5:**
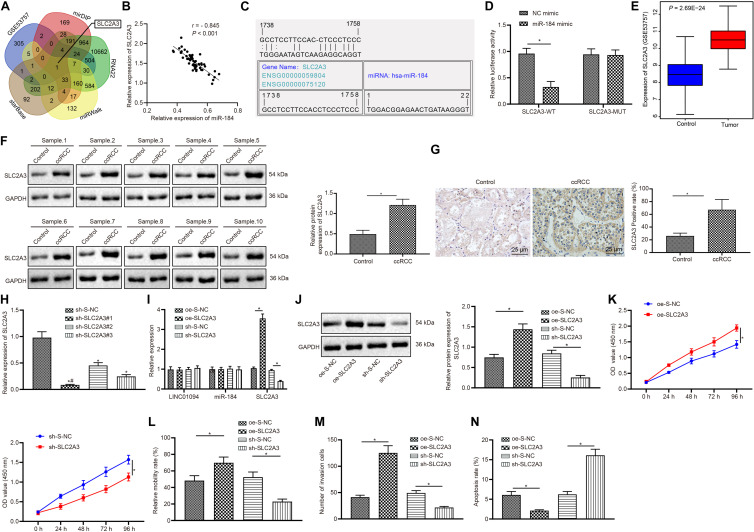
SLC2A3, a target of miR-184, regulated the development of clear cell renal cell carcinoma (ccRCC). **(A)** Bioinformatics prediction on target genes of miR-184. **(B)** Analysis of the correlation between miR-184 and SLC2A3. **(C)** RNA22 prediction on the target gene of miR-184. **(D)** The targeting relationship between SLC2A3 and miR-184 verified by dual luciferase reporter assay. **(E)** Microarray-based analysis on the expression of SLC2A3 in ccRCC. **(F)** Western Blot analysis on the protein levels of SLC2A3 in clinical ccRCC and adjacent normal tissues. **(G)** Expression of SLC2A3 in clinical ccRCC and adjacent normal tissues detected by Immunohistochemistry (400 ×). **(H)** The silencing interference efficiency of SLC2A3 evaluated by reverse transcription-quantitative polymerase chain reaction (RT-qPCR). **(I)** The expression of LINC01094, miR-184, and SLC2A3 in cells of each transfection group tested by RT-qPCR. **(J)** The protein expression of SLC2A3 analyzed by Western Blot analysis. **(K)** The viability of ccRCC cells detected by CCK-8. **(L)** The migration ability of ccRCC cells accessed by scratch test. **(M)** The invasion ability of ccRCC cells evaluated by Transwell assay (200 ×). **(N)** The apoptosis of ccRCC cells in each group analyzed by flow cytometry. * *p* < 0.05 *vs.* the NC mimic group or Control group or oe-S-NC group or sh-S-NC group, # indicated the cell line with the lowest SLC2A3 expression. The data are measurement data and expressed as mean ± SD. Paired *t*-test is performed for comparison between ccRCC tissues and adjacent normal tissues. One-way ANOVA is used to compare data among multiple groups, followed by Tukey’s *post hoc* tests. Data at different time points are compared using two-way ANOVA. Pearson correlation analysis is used to observe the correlation of indicators.

The target genes of miR-184 were predicted in mirDIP, RNA22, miRWalk, and starBase, and there were 1604, 13380, 988, and 573 potential target genes obtained, respectively. These genes were then intersected with the differentially expressed genes obtained from the microarray dataset GSE53757, accompanied by the plotting of Venn diagrams (Figure 5A). As a result, there was only one intersected gene SLC2A. Pearson’s correlation analysis (Figure 5B) showed that the level of miR-184 in ccRCC tissues was negatively correlated with that of SLC2A3 (all *p* < 0.0001) and it was speculated that miR-184 could regulate SLC2A3 expression. Furthermore, according to RNA22, we found the binding site between miR-184 and SLC2A3 (Figure 5C). Meanwhile, the dual-luciferase reporter assay was conducted to verify the binding relationship between miR-184 and SLC2A3. The results showed that the luciferase activity of the WT-SLC2A3 was decreased by miR-184 mimic (*p* < 0.05), while no significant difference in luciferase activity of the MUT-SLC2A3 (*p* > 0.05) was observed, indicating that miR-184 could specifically bind to the SLC2A3 gene (Figure 5D). Furthermore, in the GSE53757 microarray dataset, the expression of SLC2A3 in ccRCC tissues was higher than that of normal controls (Figure 5E). Additionally, the results of western blot confirmed the higher protein levels of SLC2A3 in ccRCC tissues compared with that in adjacent normal tissues (*p* < 0.05; Figure 5F). Results of immunohistochemistry showed that SLC2A3 protein, which showed brownish yellow, was distributed in the cytoplasm and nucleus, and the positive expression of SLC2A3 in ccRCC tissues was much higher than that of adjacent normal tissues (*p* < 0.05; Figure 5G). The above-described results indicated that SLC2A3, a target gene of miR-184, was up-regulated in ccRCC.

Furthermore, three silent interference sequences for SLC2A3 were designed followed by the detection of silencing efficiency by RT-qPCR. The sh-SLC2A3#1 sequence with the lowest levels of SLC2A3 was selected for subsequent experiments (all *p* < 0.05; Figure 5H). After that, the ccRCC cells were transfected with oe-S-NC, oe-SLC2A3, sh-S-NC, and sh-SLC2A3 plasmids, respectively. Then the expression of LINC01094, miR-184, and SLC2A3 in the cells of each group was detected by RT-qPCR and Western blot analysis. The results showed that there were no significant differences in the levels of LINC01094 and miR-184. When compared with oe-S-NC, the transfection with oe-SLC2A3 increased the expression of SLC2A3, while sh-SLC2A3 led to the opposite tendency of SLC2A3 expression in comparison to sh-S-NC (all *p* < 0.05; Figures 5I,J). CCK8, scratch test, Transwell assay, and flow cytometry were used to detect the growth, migration, invasion, and apoptosis of ccRCC cells in each group. It was found that overexpression of SLC2A3 promoted the growth, migration, and invasion of 786-O cells but restrained cell apoptosis, while knockdown of SLC2A3 inhibited the growth, invasion, and migration of 786-O cells but enhanced the cell apoptosis (all *p* < 0.05; Figures 5K–N). The above-described results indicated that overexpressed SLC2A3 promoted the proliferation, migration, and invasion of ccRCC cells, but inhibited the cell apoptosis.

### miR-184 Down-Regulated SLC2A3 Expression to Prevent ccRCC Development

**FIGURE 6:**
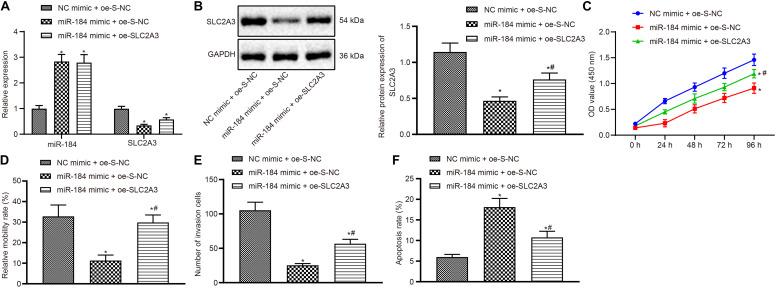
MiR-184 prevented the development of clear cell renal cell carcinoma (ccRCC) by inhibiting SLC2A3. **(A)** The expression of miR-184 and SLC2A3 in the cells of each group detected by reverse transcription-quantitative polymerase chain reaction (RT-qPCR). **(B)** The protein expression of SLC2A3 in the cells of each group analyzed by Western blot. **(C)** The viability of ccRCC cells in each group evaluated by cell counting kit-8 (CCK8). **(D)** The migration ability of ccRCC cells in each group accessed by scratch test detection. **(E)** The invasion ability of ccRCC cells in each group tested by Transwell detection (200 ×). **(F)** The apoptosis of ccRCC cells in each group measured by flow cytometry. * *p* < 0.05 *vs.* the NC mimic + oe-S-NC group. # *p* < 0.05 *vs.* the miR-184 mimic + oe-S-NC group. The data are measurement data and expressed as mean ± SD. One-way ANOVA is used to compare data among multiple groups, followed by Tukey’s *post hoc* tests. Data at different time points are compared using two-way ANOVA.

The cells were transfected with NC mimic + oe-S-NC, miR-184 mimic + oe-S-NC, and miR-184 mimic + oe-SLC2A3 plasmids, respectively. After that, RT-qPCR was performed to detect the levels of miR-184 and SLC2A3 in cells of each group. Compared with the cells treated with NC mimic + oe-SLC2A3-NC, other treatments caused significantly reduced SLC2A3 levels but up-regulated miR-184 levels (all *p* < 0.05; Figure 6A). Then the expression of SLC2A3 in the cells co-treated with miR-184 mimic and oe-SLC2A3 was increased but no significant difference was observed in miR-184 expression (all *p* < 0.05; Figure 6A). The results of the Western blot assay further confirmed the results of RT-qPCR (all *p* < 0.05; Figure 6B). The proliferation, migration, invasion, and apoptosis of ccRCC cells were evaluated using CCK8 assay, Scratch test, Transwell assay, and flow cytometry, respectively. The results displayed that up-regulating miR-184 inhibited the proliferation, migration, and invasion but promoted apoptosis of 786-O cells. However, the transfection of oe-SLC2A3 reversed the effects of miR-184 mimic (all *p* < 0.05; Figures 6C–F). The above-described results suggested that overexpressed miR-184 could inhibit the expression of SLC2A3, thereby inhibiting the proliferation, migration, and invasion of ccRCC cells but promoting cell apoptosis.

### LINC01094 Promoted the Development of ccRCC *in vivo*

**FIGURE 7:**
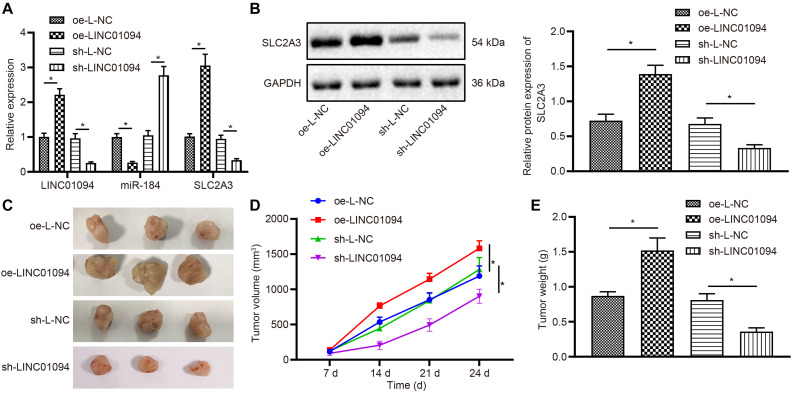
LINC01094 promoted clear cell renal cell carcinoma (ccRCC) development *in vivo.* **(A)** The expression of LINC01094, miR-184, and SLC2A3 in nude mice tumor tissue of each group detected by reverse transcription-quantitative polymerase chain reaction (RT-qPCR). **(B)** The expression of SLC2A3 protein in nude tumor tissue cells of each group analyzed by Western blot assay. **(C)** Tumor anatomy of nude mice in each group. **(D)** The growth curves of tumor volume of nude mice in each group. **(E)** The tumor weight of nude mice in each group. * *p* < 0.05 *vs.* the oe-L-NC group or sh-L-NC group. The data are measurement data and expressed as mean ± SD. One-way ANOVA is used for data comparison among multiple groups, followed by Tukey’s *post hoc* tests. Tumor volume data at multiple time points were compared using repeated-measures ANOVA, followed by Bonferroni’s *post hoc* test.

To test the effect of LINC01094 on xenograft tumors in nude mice, the xenograft models were successfully established. RT-qPCR results showed that compared with the mice treated with oe-L-NC, the expression of LINC01094 and SLC2A3 was increased in the tumors of mice treated with oe-LINC01094, while the expression of miR-184 was decreased. In comparison to sh-L-NC, the treatment with sh-LINC01094 down-regulated the levels of LINC01094 and SLC2A3 but up-regulated that of miR-184 (all *p* < 0.05; Figure 7A). According to Western blot, in the tumor tissues from mice injected with cells that were transfected with oe-LINC01094, the protein levels of SLC2A3 were increased while sh-LINC01094 led to the opposite tendency (all *p* < 0.05; Figure 7B). The measurement on tumor mass and volume showed that overexpression of LINC01094 could promote tumor growth (Figures 7C,D) and tumor weight (all *p* < 0.05; Figure 7E). However, silencing LINC01094 caused the opposite effects. The finding suggested that overexpression of LINC01094 promoted the development of ccRCC *in vivo*.

## Discussion

Clear cell renal cell carcinoma causes more than 80% of renal malignancies (Wu et al., 2018) and reported to be most prevalently diagnosed subtype with more aggressive symptoms than papillary and chromophobe RCC (Lee et al., 2018). Moreover, ccRCC is a complex disease and its molecular mechanism underlying metastasis remained unclear (Yang et al., 2019). Therefore, unraveling its underlying molecular mechanism is urgently required. In the present study, we aimed to find a novel therapy for ccRCC and verified that down-regulating LINC01094 could increase miR-184 expression and inhibit SLC2A3 expression, thereby curtailing the development of ccRCC.

First of all, we proved that LINC01094 was highly expressed in ccRCC tissues and cells, and silenced LINC01094 inhibited the proliferation, migration, and invasion of ccRCC cells but promoted the cell apoptosis. Consistent with our findings, it has been reported that LINC01094 plays its pro-tumor role in the development of ccRCC by regulating the miR-224-5p/CHSY1 axis (Jiang et al., 2020). Whilst, lncRNA PVT1 has been indicated to function as a tumor promoter that has a significant role in the metastasis in ccRCC (Bao et al., 2017). Moreover, Jiang et al. (2020) have reported that LINC01094 was activated by FOXM1 at the transcriptional level and their experimental indicated that LINC01094 acts as a sponge of miR-224-5p while CHSY1 was a miR-224-5p-targeted mRNA indicating the potential value of miR-224-5p/CHSY1 regulatory axis. Accordingly, the present study attempted to further investigate the role of LINC01094 in promoting the development of ccRCC by up-regulating the SLC2A3 via miR-184.

Besides, we demonstrated that miR-184 was a target of LINC01094, and silencing LINC01094 could increase the expression of miR-184 in ccRCC cells. Likewise, lncRNA UCA1 has been reported to promote the development of cardiac hypertrophy by binding miR-184 (Zhou et al., 2018). Whilst lncRNA MIAT possesses the ability to promote the proliferation and cisplatin resistance of non-small cell lung cancer cells via decreasing the miR-184 expression (Wu et al., 2020). Consistently, our data illustrated the down-regulation of miR-184 expression in the ccRCC tissues and cells. On the other hand, up-regulating miR-184 could inhibit the proliferation, migration, invasion of 786-O cells and tumor growth, but promoted its apoptosis. Consistently, the role of miR-184 has been documented in the development of ccRCC (Huang et al., 2016). According to previously reported research, miR-184 in renal cell carcinoma cells was significantly lower than that in normal adjacent tissues (Leng et al., 2011). Moreover, miR-184 functions as a tumor suppressor in renal cell carcinoma where it suppresses cell proliferation and induces renal cancer cell apoptosis (Su et al., 2015).

Intriguingly, the present study identified that SLC2A3 is a target gene of miR-184 and is highly expressed in ccRCC tissues and cell, whereas, the down-regulation of SLC2A3 was associated with the ccRCC development. Similarly, a previous study has indicated the high expression of SLC2A3 in the samples of ccRCC (Sanders and Diehl, 2015). Besides, SLC2A3 genes have been reported to exhibit high expression in patients with colorectal cancer (Kim et al., 2019). Another study has described the high expression of SLC2A3 in patients with papillary thyroid carcinoma, which is closely related to increased mortality (Chai et al., 2017). Thus these above described findings support our data that SLC2A3 is a target gene of miR-184 and is highly expressed in ccRCC tissues and cells. Furthermore, highly expressed miR-29c can inhibit prostate cancer cell proliferation by inhibiting SLC2A3 expression (Li et al., 2018). miR-129-5p inhibited the glycolysis and proliferation of gastric cancer cells by inhibiting the SLC2A3 (Chen et al., 2018). The deletion of lncRNA NICI can curtail the hypoxic induction of SLC2A3 (Lauer et al., 2020).

Hence, on the basis of the above findings, our study proved that silencing LINC01094 inhibited the SLC2A3 expression by up-regulating miR-184, which further inhibited the proliferation, migration, and invasion of 786-O cells and tumor growth but promoted apoptosis. The identification of the LINC01094/miR-184/SLC2A3 axis in the progression of ccRCC may aid with a better understanding of the mechanisms of ccRCC, as well as with potential of serving as a target for ccRCC treatments in the future. In the current study, we indicated that LINC01094 was mainly located in the cytoplasm of ccRCC cells, while we did not detect the localization of the lncRNA in tumor sample, which is also our limitation. This study is a preliminary trial and we will continue to verify for a clinical study.

## Data Availability Statement

The datasets generated and/or analyzed during the current study are available from the corresponding author on reasonable request. The ccRCC-related microarray data were obtained from the Gene Expression Omnibus (GEO) database (https://www.ncbi.nlm.nih.gov/geo/), GSE53757 (miRNA), and GSE53757 (gene).

## Ethics Statement

The specimens collected in this experiment were approved by Nantong Tumor Hospital, and the patients signed the Informed Consent for Specimen Disposal before the operation to voluntarily provide the kidney tissue required for this experiment.

## Author Contributions

HX, XW, and JH designed, edited, and reviewed the manuscript. HX, JW, and HJ contributed to data collection, data analysis, and experiments. ZC and HG prepared the figures. All authors contributed to the article and approved the submitted version.

## Conflict of Interest

The authors declare that the research was conducted in the absence of any commercial or financial relationships that could be construed as a potential conflict of interest.

## Footnotes

1 https://www.ncbi.nlm.nih.gov/geo/

2 http://lncatlas.crg.eu/

3 https://cm.jefferson.edu/rna22/

4 http://ophid.utoronto.ca/mirDIP/

5 http://mirwalk.umm.uni-heidelberg.de/

6 http://starbase.sysu.edu.cn/index.php

7 http://bioinformatics.psb.ugent.be/webtools/Venn/

8 http://www.cellbank.org.cn

## Supplementary Material

FIGURE S1 LINC01094 promotes clear cell renal cell carcinoma (ccRCC) progression by inhibiting miR-184 expression in 769-P cell line. **(A,B)** The expression of LINC01094 and miR-184 measured using reverse transcription-quantitative polymerase chain reaction (RT-qPCR). **(C,D)** The cell viability of ccRCC cells measured using cell counting kit-8 (CCK8). **(E,F)** The migration ability of ccRCC cells examined by scratch test. **(G,H)** The invasion ability of ccRCC cells in each group detected by Transwell assay (200 ×). **(I,J)** The apoptosis of ccRCC cells in each group accessed by flow cytometry. ^∗^*p* < 0.05 *vs.* the NC mimic group or NC inhibitor group or sh-L-NC + NC inhibitor group, # *p* < 0.05 *vs.* sh-LINC01094 + NC inhibitor group. The data are measurement data and expressed as mean ± SD. Paired *t*-test is performed for comparison between cancer tissues and adjacent normal tissues. One-way ANOVA is used to compare data among multiple groups, followed by Tukey’s *post hoc* tests. Data at different time points are compared using two-way ANOVA.
