# Supplementary figures and images for: Long Non-coding RNA LINC01094 Promotes the Development of Clear Cell Renal Cell Carcinoma by Upregulating SLC2A3 via MicroRNA-184
Source: Front Genet. 2020 Sep 23;11:562967. doi: 10.3389/fgene.2020.562967 (PMC7538661; doi:10.3389/fgene.2020.562967)

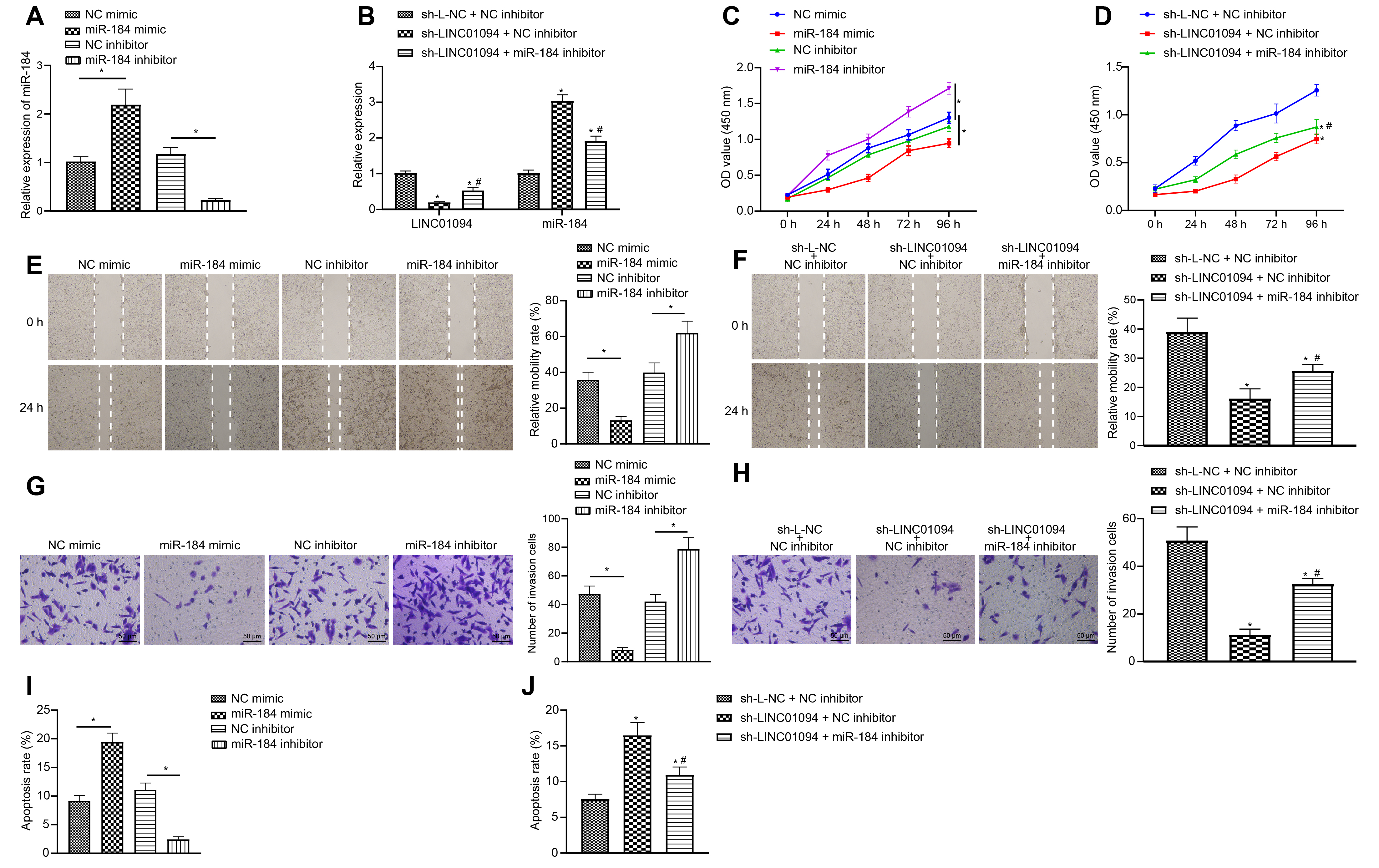

Supplement: FIGURE S1 — LINC01094 promotes clear cell renal cell carcinoma (ccRCC) progression by inhibiting miR-184 expression in 769-P cell line. (A,B) The expression of LINC01094 and miR-184 measured using reverse transcription-quantitative polymerase chain reaction (RT-qPCR). (C,D) The cell viability of ccRCC cells measured using cell counting kit-8 (CCK8). (E,F) The migration ability of ccRCC cells examined by scratch test. (G,H) The invasion ability of ccRCC cells in each group detected by Transwell assay (200 ×). (I,J) The apoptosis of ccRCC cells in each group accessed by flow cytometry. ∗p < 0.05 vs. the NC mimic group or NC inhibitor group or sh-L-NC + NC inhibitor group, # p < 0.05 vs. sh-LINC01094 + NC inhibitor group. The data are measurement data and expressed as mean ± SD. Paired t-test is performed for comparison between cancer tissues and adjacent normal tissues. One-way ANOVA is used to compare data among multiple groups, followed by Tukey’s post hoc tests. Data at different time points are compared using two-way ANOVA. [file Image_1.jpg]
