# Supplementary material for: Long Non-coding RNA LINC01094 Promotes the Development of Clear Cell Renal Cell Carcinoma by Upregulating SLC2A3 via MicroRNA-184
Source: Front Genet. 2020 Sep 23;11:562967. doi: 10.3389/fgene.2020.562967 (PMC7538661; doi:10.3389/fgene.2020.562967)
Supplement: Supplementary file 2 [file Table_1.DOCX]

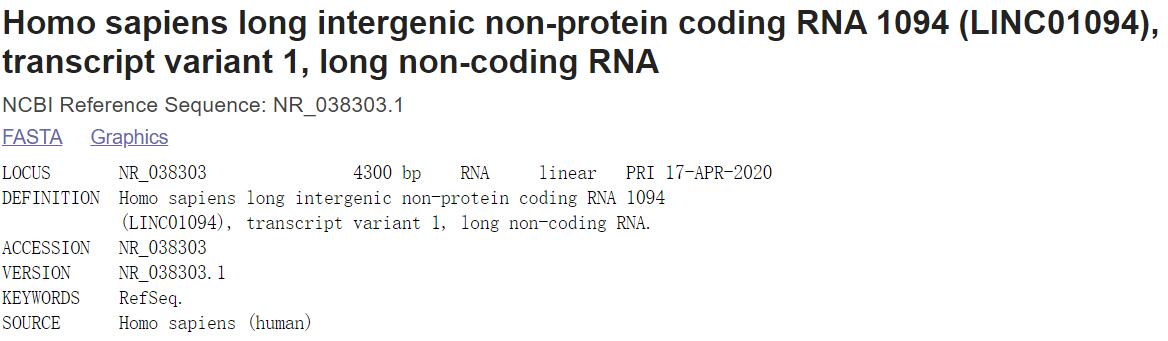


cttttcagtt ctgtcttcct gaggctgctg tttctgggca tggccagggg atcttcagac

61 tttacaaacc agttgtgcag agccaggcat ttcaataagg aacaaaatgc tgagtaacaa

121 catgaagtca gagtgttgct tgccaagagc tgggaggcct gggtggtttc ccaatcttaa

181 agaagcagaa ggatattaaa gtgagcctcc caaaggaaaa cgtggatcta cttaccaacg

241 cagaaaatga tacacacgga ttcgatttag gcacgctaca ttcaaatgac ttggaagagc

301 aggttctctg agccaggata ctaaagtccc tgtttaggaa agagccacgt tgtatgttta

361 ccctaccaca aagatacagt catgtgcctg ggaaagattg atgccagctc cagaaaggca

421 tccgaatggt ctaagtaatg ccatccatct gtcagtaata gattcagaaa tggtgctggc

481 agaggaaaca gcagcaatga tgctctgcga gcctgactca gggccagcag tgatggcagc

541 ccaagaattc tggaggaaag tggctctggg aattgaacag tggctcagtg atgttggggc

601 caccaagtct gcaattctcc ctacctgttc ttcatggttg taagatgact gctgcaattc

661 tcggcatcat gtgcatgtta atgtcaggag gaagaaagga agagggagca ctgggatgtt

721 acattcattt tgtcaggaaa agcaaagcct ttccagacag ctccgaaact tgcttacctc

781 tcattggcca gcaccatcac ttgaccactt gtccactcct agctgcaagg aaagtggcaa

841 agtagaaggt atttgagaat gtctgctggg ttagccaaaa caatagtgac ttctacaccc

901 aattttctgg gcactgcaga agtatccttg agtcctatgt gatcagaggg agagcaaacc

961 tcaataatga aacagattca atttccatca cccagtgaga caccatggga ttagaataaa

1021 tttttaaaaa aacaaattga aaaaaatgca tttttaaaac aacaaaatca gaaaggatga

1081 aaggtgatat ttctagcata tcagtttgtg gtggtaaatt aattacctta tacaaaggcc

1141 tcagtgactt tcttctcctt acctaacgaa tcatctttaa aataagtaaa ataaagaaac

1201 aagagcagca gcagatgctt cctaaaaaga agttaccaaa tctgttcacc acaaacctga

1261 gaattcaact gtggacattt ggacaccagc aatgaacatg aaactcagct tatgaggcat

1321 atttggctaa aggtcaaggt gacttaaagt tcatcatagg acatctacaa tttgcaaagc

1381 aggtgactgg atatggactc cttccaagtc tcatttaatt ggataatgcc tacgttttaa

1441 gtatcagaag tgagtctttc tctattaaat gaagtcagtt ccactaaaac ctaaatcttg

1501 tttggcaggc actccatgtt tcttaatttt tattccatgc tgtatattct tttagacttg

1561 attttcactt ttttttcttt tttcctgtac atttatcttt gctgtgaaac aaagtgccct

1621 gctttgaagg acaggtttgt gggctgctgg tggtgagaca acagcagtcc ttccaagtct

1681 tcctcccatc actctgttag ctctcccttt gtttttaatt agcattacac cagttaattc

1741 tgttatctct taaccctggt tctgcatcct cttcttcttt tctattcctt actccgctcc

1801 tgttttttct tgttctgctt ttccaagaaa tgtcttgtct cctcatcagt tgactgtctt

1861 ctctttccag agggaccata aaggtgccag cctaaggaac acgtaaaacg tgggaacaga

1921 caaacaagct gctgcctatt acagagatag ataattaact ttggaccatt acatgcaatt

1981 catcagctta tctttttcaa actttatata agaagtcatt tcccaatggt aaccacttgc

2041 cagctgcaac tttgacgcat attatcagga taggtgagat tgatgggggc cctttgaact

2101 cttcccaggg aattatcttt ataatgttac agttttctct atagcatggt catattgtta

2161 acattggtta taaggtcata tggttagtat ggttagtatt ggtggctcac agctgtaata

2221 ccagcacttt gggaggccaa ggtgggagga tcacttgagc cctggtgttc aaaaccagct

2281 ctggaaacat agcaagacct tgtctctaca aaaaaaaatt aaataaatta aaagattagc

2341 tgggcatggt ggcacatgcc tgtagtcctc actacttggg aaactaaggt gggaggatcc

2401 tttgagcttg ggaggttgag gctgcagtga gctgtggttg tgccatggca ctccagcctg

2461 ggtgacagag tgagagcctg tctcaaaaaa aaaaaaaaaa aaaaaaaaaa gtacctgtaa

2521 ggtctttggg attttgaaga tgaaaggtgt tgcataaatt gaatgaattc acttagagag

2581 atagaaggca actacaggga atattacgta catcatggag cggaaagcac taaggcctgt

2641 ggaattattc tcagtgacac gtcgttgagg gcagtggtcc acatgttttg aacatggttt

2701 ctcccaggtg aaaaggatga gtaatttgtt ctccttcaca tgtgtgtatg taggtcttat

2761 acacagcaga tttgccacaa aacaaatgaa gattacactt tgggcccctc tctcacatga

2821 gccccttcca aagttctgtt tgctaatttt acattttgta atttcttatt atttttctta

2881 aagaagcctc ctccaattgc ataaacttca agcctcacaa aatttggatc catacctgag

2941 ccaacataca atacaatttt ccatttctaa cttttagttg tacctagtgt tttattggaa

3001 atgatcaagt acatgagtgg tgtcttttta aaaaatttac tttatttgct tctctagctt

3061 catcgttagt taggcagtta aatctaacca tttggaggta ttttcatata acctccattg

3121 aggacaattt ggcaatagct tttaaaatta taaatatatt tgtacagtta taagctgcca

3181 tatctataaa actatttgat agacaaaagt tgaaaaccct ccaagagctc atcaataaaa

3241 gattaaatta attatgatac atccatatga gcactgagca gctgtgaaaa aaatgaggaa

3301 gtaattcctg tgctgatatg ggaagacttc caagacatgt tgttaagtac agaatggtgt

3361 atacagtatg ctatatagtg tggtaaaagg tttagggata agcatttata tttataactg

3421 tttatattta cataaagtaa ttctgaaagg atacaaaaaa gctaacaaaa gagattacct

3481 gtggtagtgt ggggcttggg gattggatgg gtgagaaaca gtagtggaaa tgagatattg

3541 tacagtttaa ggcattatac acctataaaa acatgggaat gtattaccta ctcaaaaagt

3601 gaaattaaga gagatacaag aaactgtttg gatgttcctt cccgtttcaa tgtatctata

3661 ttttttcagc cctcaaagtt attataaatg tcattataat aacttcataa agcttcccct

3721 ataatgagaa tcttaccatt ctcctaactc caatagcact gtaatccacc tctgttagga

3781 cacatcactc ttcatcatgt gtgattatta tttgcagata tcatcttcaa ggctcttctc

3841 tatttaccac ctgccattct tgttctcacc tctacatgac cttcacattt tatttctctc

3901 ggttttttgg acaagctaca ttcccttcca cagagaaggc ttccctgacc cctttcactt

3961 ggttggatta ccttgctatt cttttctatc tttgtacatt ccttttcaaa atacttatct

4021 cccttgttgt tacctataat gtctgtcttc tttactcaag agtctagaca aaaagctcca

4081 cgccagcctc ggctgtgttt gtattgttta cgtctgtgtt cctagtgctt aataaaatac

4141 ctagcaaggg ttaataagta cttgttgact ggaagggaat gacttccttc ccttactaga

4201 gagtgatttc cttacaggcg agatgtgtca accttctata gcccacataa aataaaggac

4261 aatatctcct attggataaa agacaaaaaa aaaaaaaaaa
